# *Neospora caninum* antibodies in dairy cows and domestic dogs from Vojvodina, Serbia

**DOI:** 10.1051/parasite/2013036

**Published:** 2013-10-24

**Authors:** Ljiljana Kuruca, Ljubica Spasojević-Kosić, Stanislav Simin, Milan Savović, Saša Lauš, Vesna Lalošević

**Affiliations:** 1 Faculty of Agriculture, University of Novi Sad Trg Dositeja Obradovića 8 21000 Novi Sad Serbia; 2 PVP MSV Medicus D.O.O. Milice Stojadinović Srpkinje 1 21209 Bukovac Serbia; 3 Praxis Veterinaria D.O.O. 2. Oktobra 40 26300 Vršac Serbia

**Keywords:** Anti-*Neospora caninum* antibodies, Dairy cows, Dogs, Risk factors, Vojvodina

## Abstract

*Neospora caninum*, the causative agent of neosporosis, is a protozoan parasite responsible for high rate of abortion in cattle worldwide. In dogs, consequences of infection vary from severe neuromuscular disorders to asymptomatic infection and shedding of environmentally resistant oocysts. In this study, we determined the occurrence of *N. caninum* antibodies in dairy cattle and dogs in Vojvodina (Northern Province of Serbia) and possible risk factors. *N. caninum* antibodies were found in 15.4% (55/356, CI 95%:12.0–19.6) of cows and 17.2% (17/99, CI 95%: 10.8–26.2) of dogs. Cows from smallholdings showed significantly greater odds (OR = 5.28, CI 95%: 2.0–13.6, *p* = 0.0006) of being seropositive in comparison to the farm cows. Epidemiological importance of results is discussed.

## Abstract (fr)

*Neospora caninum*, l’agent qui cause la néosporose, est un parasite protozoaire responsable d’un taux élevé d’avortements dans le bétail à travers le monde. Chez les chiens, les conséquences de l’infection varient entre un trouble neuromusculaire sévère et une excrétion asymptomatique d’oocystes résistants dans l’environnement. L’objectif de cette étude était d’examiner le statut sérologique des vaches laitières et des chiens en Voïvodine (province du nord de la Serbie) et les facteurs de risque possibles. Des anticorps contre *N. caninum* ont été détectés dans 15,4 % (55/356, CI 95 %:12,0–19,6) des vaches et dans 17,2 % (17/99, CI 95 %:10,8–26,2) des chiens. Les vaches des petits propriétaires ont manifesté une probabilité significativement plus importante (OR = 5,28, CI 95 %:2,0–13,6, *p* = 0,0006) d’être séropositives que les vaches de fermes. L’importance épidémiologique de ces résultats est discutée.

## Introduction

The protozoan parasite *Neospora caninum* causes considerable economic losses to cattle industry worldwide [1].

One of the definitive hosts of *N. caninum*, which is responsible for shedding of environmentally resistant oocysts, is the domestic dog [6]. In dogs, neosporosis can cause severe clinical manifestations, including myositis-polyradiculoneuritis, encephalomyelitis and dermatitis [7, 12].

Worldwide reports of clinical and subclinical infections in all hosts were summarized by Dubey et al. [1] and Dubey and Schares [2], but there was no mention of neosporosis in Serbia. Recently, occurrence of *N. caninum* specific antibodies in both cattle and dogs has been confirmed in Serbia as well [3, 9, 13, 17] and published in local journals. The aim of this study was to investigate current serological status of dairy cattle and dogs in Vojvodina (Northern Province of Serbia), with regard to the possible risk factors.

## Material and methods

### Studied area

Vojvodina is a northern (45°15′ N 19°50′ E) province of the Republic of Serbia which occupies 21,506 km^2^ of the state territory [5]. The major part of the province’s territory consists of fertile plains with the Danube, Tisa and Sava rivers dividing it in to three regions: Bačka, Banat and Srem. The climate of Vojvodina is moderately continental, characterised by hot, dry summers, cold winters and relatively low rainfall.

### Animals and sample collection

**Figure 1.**
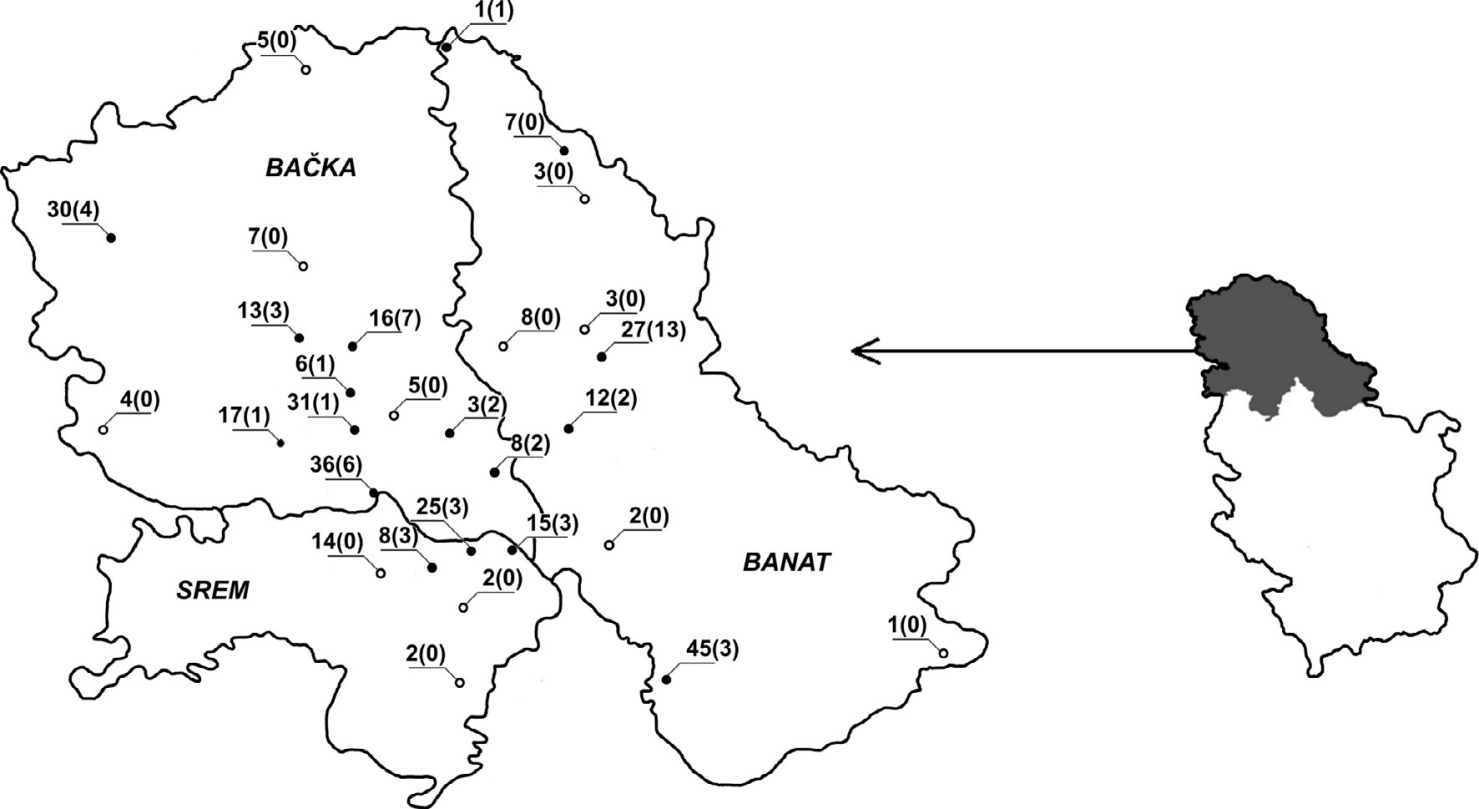
Dots on the map of Vojvodina represent localities where blood samples were taken from cows or, in the case of blood collected at the abattoir, places of cows’ origin. Black dots refer to localities where seropositive cows were found, while white dots stand for localities where there were no positive samples. Figures represent the number of sampled cows from specific locality and number of positive ones (between parentheses).

Blood samples were collected from 356 dairy cows from both commercial farms (109 cows) and smallholdings (247 cows) in Srem, Banat and Bačka region (Figure 1), during the 2009–2013 period. Samples from 271 cows were obtained on the farm by jugular venipuncture and 85 samples were collected at the abattoir. Among these were 74 samples from cows with a history of various reproductive disorders and 197 samples from reproductively healthy cows. Medical history could not be obtained for 85 abattoir samples. Prior to sampling, minimum recommended size of the sample was calculated, using Win Episcope 2.0 software [16]. Announcement of the Statistical Office of the Republic of Serbia on the number of cattle [14] and expected prevalence of 17.3% [13] that would ensure a 95% confidence interval and produce an error of 5% were used as input data for this calculation. Minimum recommended size of 220 animals was obtained. Therefore, a sample of 356 animals would not only provide an unbiased estimation of the prevalence of *N. caninum*-specific antibodies in the dairy cows of Vojvodina but would also reduce the error to 3.92%.

**Figure 2.**
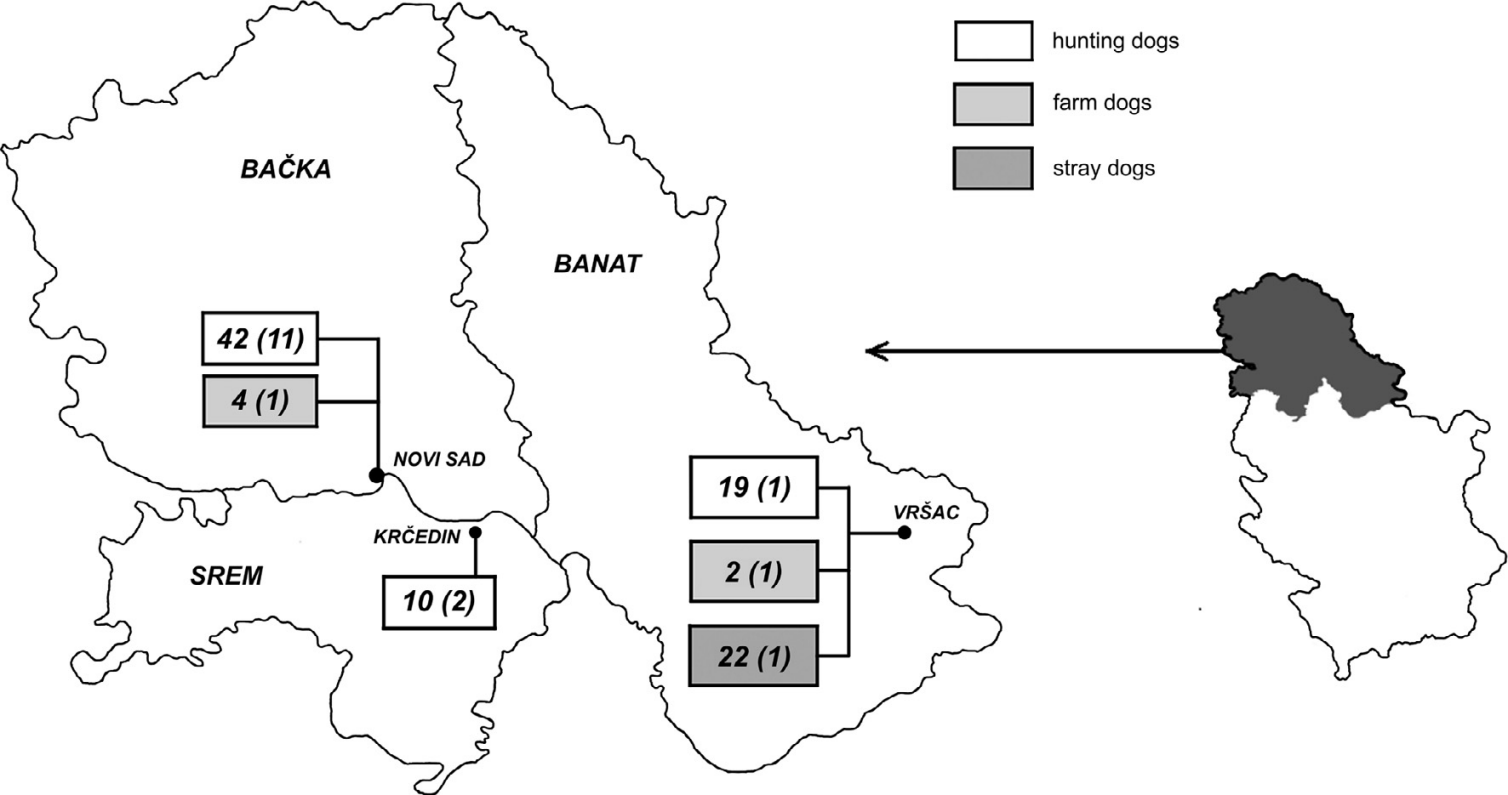
Geographical distribution of localities where blood samples were collected from hunting, farm and stray dogs. Numbers outside parentheses represent the dogs from the specific category that was tested on the given locality. Numbers between parentheses refer to the positive ones.

Blood samples were obtained from 99 dogs during the 2008–2013 period, from various sources on several locations in Srem, Banat and Bačka (Figure 2). None of the sampled dogs, including six farm dogs, belonged to the same farms as cows from this study. All canine blood samples were collected from cephalic vein. The dogs were clinically examined prior to sampling and for each dog a record sheet, which included information on breed, gender, age, previous health problem and diet, was filled. For stray dogs, only gender and age (estimated by competent veterinarian) could be obtained.

Sample preparation procedure was identical for both cows and dogs: after being left to clot, blood was centrifuged (3000 rpm for 10 min) and separated sera were stored at −20 °C until examination.

### Serological testing

Two types of serological tests were used to detect *N. caninum* antibodies in cow sera. Initially 100 sera were assayed using the commercial competitive ELISA test kit (cELISA, VMRD Inc., Pullman, USA). The remainder 256 cow and all dog sera were examined with an indirect fluorescent antibody test (IFAT) using reagents marketed by VMRD. Both tests were performed according to the manufacturer’s instructions. Cow sera, examined with ELISA, that presented inhibition percentages equal to or higher than 30% were considered positive. For IFAT, a recommended cut-off of 1:200 for cows and 1:50 for dogs was used. Dog sera that exhibited positive reaction at 1:50 were serially diluted until negativity was reached.

### Statistical analysis

Seroprevalences and their confidence intervals, for both cows and dogs, were calculated using Quantitative Parasitology 3.0. [11]. For the statistical analysis of the possible effects of different factors (origin, farm type and history of reproductive disorders in cows and utilisation, breed, gender, age, origin and feeding habits in dogs) on the occurrence of anti-*N. caninum* antibodies the chi-squared test was used at a significance level of 95% (*p* ≤ 0.05). Where appropriate chi-squared test was replaced by Fisher’s exact test or unconditional exact test. All these tests were computed using the above mentioned statistical software. Odds ratio (OR) was calculated using Win Episcope version 2.0 software [16]. Positive OR (OR > 1), with 95% confidence interval (CI) that does not overlap with the null value of 1 and with *p* ≤ 0.05, was considered statistically significant [15]. Confidence interval and *p*-value for OR were calculated using online MedCalc software version 12.6 [8].

## Results

Antibodies to *N. caninum* were found in 15.4% (55/356, CI 95%:12.0–19.6) of cow sera. Seven positive sera were detected by ELISA and the remaining 48 by IFAT (Table 1). Among the risk factors evaluated, only cows originating from smallholdings had significantly greater odds (OR = 5.28, CI 95%: 2.0–13.6, *p* = 0.0006) of being seropositive in comparison to the group of farm cows.

**Table 1.** Prevalence of seropositive cows according to the geographical origin, farm type and health status.

	Examined			Positive					
	ELISA	IFAT	Total	ELISA	IFAT	Total	Rate (%)	CI 95 (%)	*p*-Value
*Geographical origin*									
Srem	10	56	66	0	9	9	13.6	7.1–24.1	0.766
Banat	31	78	109	3	16	19	17.4	11.4–25.6	
Bačka	59	122	181	4	23	27	14.9	10.3–20.9	
*Farm type*									
Commercial farms	33	76	109	1	4	5	4.6	1.5–10.4	0.0001^a^
Smallholdings	67	180	247	6	44	50	20.2	15.4–25.8	
*Reproductive disorders*									
Yes	22	52	74	0	9	9	12.2	6.3–21.5	0.148
No	0	197	197	0	37	37	18.8	13.9–24.8	
nd	78	7	85	7	2	9	10.6	5.5–19.2	
Total	100	256	356	7	48	55	15.4	12.0–19.6	

nd = No data regarding medical history could be obtained

a Difference between these prevalences was statistically significant (*p* ≤ 0.05).

*N. caninum* antibodies were found in 17.2% (17/99, CI 95%: 10.8–26.2) of dogs, with titres of 50 in 15 dogs, 100 in two and 200 in one dog. Out of 17 seropositive animals, 14 (14/71, 19.7%, CI 95%: 11.2–30.9) came from the group of hunting dogs, one (1/22, 4.5%, CI 95%: 0.2–22.2) was a stray dog and two (2/6, 33.3%, CI 95%: 6.3–72.9) belonged to a small group of farm dogs. Of all the risk factors evaluated statistical difference (*p* = 0.02) was observed in the occurrence of *N. caninum*-specific antibodies in dogs from Bačka versus dogs from Banat (Table 2).

**Table 2.** Prevalence of anti-*Neospora caninum* antibodies in hunting, stray and farm dogs according to the breed, gender, age and region of origin.

	Hunting			Stray			Farm			Total		
	*n*	Positive	%	*n*	Positive	%	*n*	Positive	%	*n*	Positive	%
*Breed*												
Purebreed	66	14	21.2	0	0	0	2	1	50	68	15	22.1
Mongrel	5	0	0	22	1	4.5	4	1	25	31	2	6.5
*Gender*												
Female	32	5	15.6	13	1	7.7	2	1	50	47	7	14.9
Male	39	9	23.1	9	0	0	4	1	25	52	10	19.2
*Age (years)*												
≤1	13	1	7.7	6	0	0	2	0	0	21	1	4.8
>1, ≤5	45	11	24.4	13	1	7.7	4	2	50	62	14	22.6
>5	13	2	1.4	3	0	0	0	0	0	16	2	12.5
*Origin*												
Srem	10	2	20	0	0	0	0	0	0	10	2	20
Banat	19	1	5.3	22	1	4.5	2	1	50	43	3	7
Bačka	42	11	26.2	0	0	0	4	1	25	46	12	26.1^a^
Total	71	14	19.7	22	1	4.5	6	2	33.3	99	17	17.2

*n* = Number of examined dogs; % = prevalence

a Difference between these prevalences was statistically significant (*p* ≤ 0.05).

In 42 hunting dogs with known feeding habits 11 (26.2%, CI 95%: 14.9–41.6) were seropositive. No statistical differences (*p* = 0.4) were found between the dogs fed commercial food (2/12, 16.7%, CI 95%: 2.1–48.4), homemade food (8/22, 36.4%, CI 95%: 17.2–59.3) and the mixture of two diets (1/8, 12.5%, CI 95%: 0.6–50.0).

Seropositive purebred dogs were present as follows: Dachshund (*n* = 1), English Springer Spaniel (*n* = 1), English Pointer (*n* = 1), German Shorthaired Pointer (*n* = 4), German Hunting Terrier (*n* = 1), Weimaraner (*n* = 1), Setter (*n* = 1), German Wirehaired Pointer (*n* = 2), Labrador Retriever (*n* = 1), Small Munsterlander Pointer (*n* = 1) and German Shepherd (*n* = 1).

Most (92 of 99) dogs were clinically normal and seven dogs had clinical signs which could not be related to neosporosis.

## Discussion

The results of this study corroborate previous findings [3, 9, 13, 17] of *N. caninum* antibodies in both cattle and dogs from the territory of Vojvodina.

The prevalence of antibodies in dairy cattle from our study (15.4%) was higher than the one found by Gavrilović et al. [3]. Similar to our study, their sample consisted of both aborting and randomly sampled cows, from both commercial and smallholding farms. Nevertheless, they found only 4.6% (23/500) of cows to be seropositive, which could be partly due to the small proportion of aborting cows in the total sample and the fact that they restricted their research to the south district of Banat. Different commercial tests were utilized in these studies (Gavrilović et al. used ELISA manufactured by IDEXX and Svanova, while we used IFAT and ELISA manufactured by VMRD) which may have also influenced the results. Vidić et al. [17] and Savović et al. [13] reported occurrence of *N. caninum* antibodies in 3.7% (5/132) and 17.3% (9/52) of dairy cows, respectively. Although small size of the study samples and the fact they consisted entirely of cows with reproductive disorders make these prevalences difficult to compare with, we referred to them in the interest of data transparency.

With regard to the risk factors, significant difference was observed between seroprevalences in cattle from commercial and from small farms in our study. Probable reason for these observations is the fact that biosecurity measures on commercial farms are generally superior to those on small family farms which are more likely to have dogs, the potential source of infection. Furthermore, cattle from smallholdings are usually associated with extensive production and often allowed to graze, which is, according to some authors [4, 10], one of the risk factors associated with seropositivity in cattle. Gavrilović et al. [3] also observed that all cows from seropositive commercial farms in their study had access to the outdoor paddocks which resulted in higher herd prevalence in commercial farms (50.0%), in comparison to smallholdings (22.64%). On individual level, however, they found no significant difference between commercial (4.67%) and small (4.50%) farms.

Prevalence of *N. caninum* antibodies in dogs varies based on many factors, including age, origin and diet [1]. Pavičić et al. [9] were first to detect *N. caninum* antibodies in four out of 31 (12.9%) dogs from Vojvodina. Examined dogs were mostly hounds, originating from Srem and Banat region. In the present study, we found antibodies in 17 out of 99 dogs (17.2%). Samples were collected from all three regions of Vojvodina and higher prevalence was observed in dogs from Bačka (26.1%) in comparison to dogs from Banat (7%). Other factors, such as breed, age, diet or utilisation did not appear significant. However, since the sample size was relatively small and samples originated from various sources and were collected over several years, no definitive conclusion can be made. Nevertheless, we recorded findings for the benefit of future studies.

Presently, there are no reports of confirmed clinical neosporosis in any host in Serbia, including cattle and dogs.

In conclusion, our study confirmed the presence of *N. caninum* antibodies in general population of dairy cows in Vojvodina, with a higher prevalence detected in animals originating from smallholding farms. Specific antibodies against *N. caninum* were also found in dogs from the same province, with slightly higher prevalence observed in dogs from Bačka.
